# VEGF Detection via Impedance Spectroscopy on Surface Functionalized Interdigitated Biosensor

**DOI:** 10.3390/jfb14070365

**Published:** 2023-07-12

**Authors:** Yue-Der Lin, Serge Ismael Zida, Chu-Chun Yang, Yit Lung Khung

**Affiliations:** 1 Ph.D. Program of Electrical and Communications Engineering, Feng Chia University, No. 100, Wenhwa Road, Seatwen, Taichung 40724, Taiwan; serge_ismael@live.fr; 2 Master’s Program of Biomedical Informatics and Biomedical Engineering, Feng Chia University, No. 100, Wenhwa Road, Seatwen, Taichung 40724, Taiwan; mark02254888@gmail.com; 3 Department of Automatic Control Engineering, Feng Chia University, No. 100, Wenhwa Road, Seatwen, Taichung 40724, Taiwan; 4 Department of Biological Science and Technology, China Medical University, No. 100, Section 1, Jingmao Road, Beitun District, Taichung 406040, Taiwan

**Keywords:** DNA aptamer, interdigitated sensor, impedance spectroscopy, vascular endothelial growth factor (VEGF)

## Abstract

Vascular endothelial growth factor (VEGF), a clinically important biomarker, often plays a key role in angiogenesis, would healing, tumor growth, lung development, and in retinal diseases. Hence, detecting and quantifying VEGF is deemed medically important in clinical diagnosis for many diseases. In this report, a simple yet highly cost-effective platform was proposed for VEGF protein detection using commercially available interdigitated sensors that are surface modified to present DNA optimally for VEGF capture. The dielectric characteristics between the fingers of the sensor were modulated by the negatively charged aptamer-VEGF capture, and the impedance was estimated using an impedance analyzer. Impedance-spectra tests were compared among pristine unmodified surfaces, functionalized monolayer surfaces, and aptamer-grafted surfaces in order to evaluate the efficacy of VEGF detection. From our results, the sensitivity experiments as conducted showed the ability of the interdigitated sensor to detect VEGF at a low concentration of 5 pM (200 pg/mL). The specificity of the functionalized sensor in detecting VEGF was further examined by comparing the impedance to platelet-derived growth factor, and the results confirm the specificity of the sensor. Finally, the Nyquist plot of impedance spectra was also presented to help data visualization and the overall performance of the device was found to be a highly suitable template for a smart biosensor for the detection of VEGF.

## 1. Introduction

Early detection of diseases is often deemed crucial as it helps to open up many avenues for successful treatment. In many diseases, the presence and quantification of biomarkers serves as an important parameter in discerning pathophysiology as well as the state of disease. Thus, clinical identification and quantification of biomarkers has emerged as an important aspect in contemporary clinical diagnosis and subsequent treatment [1]. Of the many biomarkers that are of clinical significance, vascular endothelial growth factor (VEGF) is one important biomarker that plays a key role in angiogenesis as well as in many physiological and pathological processes such as wound healing [2], tumor growth [3,4,5], and lung development [6]. In addition, VEGF is often implicated in various retinal diseases, such as age-related macular degeneration (AMD) [7], diabetic retinopathy [8], and retinopathy of prematurity [9]. As such, these aspects have rendered VEGF as an important biomarker of serious clinical implications. So far, enzyme-linked immunosorbent assay (ELISA) is the main immunoassay technique for detecting VEGF under a clinical setting. The sensitivity of ELISA is well-established and can even discern the various isoforms of VEGF commonly detected during medical diagnosis [10]. However, there are some disadvantages of using ELISA for VEGF detection. Firstly, ELISA may sometimes produce false-positive or false-negative detection, and this may adversely affect the diagnosis outcome or treatment. Secondly, ELISA can be rather time-consuming and not very cost-effective for undeveloped rural areas or in some third-world countries. There are also concerns pertaining to limitation in the dynamic range of ELISA detection as well as the susceptibility of cross-interference from biological serums. Hence, it is necessary to find a quick and inexpensive replacement for ELISA, and in recent years, there has been much advancement towards developing new such diagnostic platforms.

There are a wide range of different types of biosensors that have been suggested as alternatives for the time-consuming ELISA. For instance, surface plasmon resonance (SPR) is one such sensitive and specific biosensor for detecting VEGF based on a relatively straightforward concept of optical sensing. Such biosensors rely on measuring changes in refractive index that occur in the vicinity of a sensor chip that had been precoated with immobilized bioreceptors (such as antibodies or aptamers) for the capture of VEGF. Upon binding, minute changes in refractive index can be registered as a shift in the angle of reflected light, which is proportional to the amount of VEGF bound to the sensor surface [11]. In addition, there are numerous electrochemical-based biosensor setups (amperometric, potentiometric, and impedimetric biosensors) proposed to replace ELISA. Amperometric biosensors measure the current produced by the oxidation or reduction of electroactive species generated by the binding of VEGF to an immobilized bioreceptor. In conjunction, potentiometric biosensors detect changes in the potential difference between two electrodes, one of which is functionalized with an appropriate bioreceptor and the other with a non-specific protein that serves as a reference. The potential change will be caused by the selective binding of VEGF to bioreceptors on the surface and will alter the detectable charge distribution at the electrode surface. Impedimetric biosensors, on the other hand, measure modulations of the electrical impedance from a solution containing the surface-immobilized bioreceptor and VEGF that is attributed to changes in the dielectric properties or charge distribution of the solution [12]. Additionally, label-free biosensors based on semiconductor technology, such as silicon nanowire (SiNW) field-effect transistor (FET), can offer unparalleled sensitivity for the detection of VEGF by measuring changes in conductivity when VEGF binds to the nanowire surface. Due to the small size of the nanowire and the high surface-to-volume ratio, this approach can detect extremely low concentrations of VEGF with high accuracy and precision [13]. These strategies as highlighted above exhibit relatively faster detection speed and are less labor intensive compared to conventional ELISA. However, the methodologies and industrial processes involved in producing these biosensors may be very sophisticated and hence not easily accessible for third-world countries.

An interesting and inexpensive alternative that requires simpler fabrication processes is interdigitated sensors. These sensors are generally described as microelectrodes that are arranged as interdigitated fingers that can be used to measure changes in impedance or conductivity of the surrounding medium [14]. With the binding of appropriate surface bioreceptors, VEGF molecules can be captured to the electrode surface, which subsequently changes the dielectric properties of the medium, which in turn changes the overall impedance. These changes in impedance measurements can then be used to produce an impedance spectrum by scanning at different frequencies, which in turn would provide an estimate of the concentration of VEGF molecules that is present in the medium [15]. Interdigitated sensors are considered to be promising for detecting VEGF owing to several advantages over alternative methods. Compared to ELISA, these label-free interdigitated sensors can offer rapid detection while eliminating the need for secondary antibodies, and this arrangement reduces the overall complexity and cost of the assay. Furthermore, interdigitated sensors can provide real-time monitoring of binding events, and this enables the dynamic measurement of VEGF concentration. Unlike SPR, interdigitated sensors may even offer higher sensitivity and a broader range of detection attributed to a larger sensing area, and a comparatively lower noise level. Furthermore, when compared to the other electrochemical biosensors such as amperometric and potentiometric biosensors, the impedance spectroscopy of an interdigitated sensor may provide detailed information on the binding kinetics and structural changes of the bioreceptor upon VEGF capture, and this may also allow for a better understanding of the interaction mechanism. Interdigitated sensors are generally easier to fabricate, due to design simplicity, and can be mass-produced at a lower cost compared to electronic biosensors such as SiNW FET while remaining robust for everyday use. In conjunction with these attributes, the non-destructive nature of impedance spectroscopy makes it potentially suitable for long-term monitoring and repeated measurements, while the resilience of the material allows for more robust handling even under less ideal conditions. Hence, it is therefore envisaged that miniaturized interdigitated sensors with impedance spectroscopy may have the potential to be integrated into portable and handheld devices for point-of-care testing [16], especially in rural settings where accessibility to healthcare devices remains a major concern.

So far, certain interdigitated sensors have already been developed for VEGF detection. For instance, Kim et al. proposed an impedance biosensor for detecting VEGF using a poly (3,4-ethylenedioxythiophene) (PEDOT)/gold nanoparticle (Au NP) composite material. The authors deposited PEDOT-Au NP composites on three different interdigitated electrode designs. The anti-VEGF antibodies were covalently immobilized on the surface of the polymer films, and the resulting composites were used to detect VEGF-165 using electrochemical impedance spectroscopy (EIS). Among different interdigitated electrode designs, the interdigitated strip shape showed the best overall film stability and reproducibility [17]. In another study, Sun et al. utilized the complementary metal-oxide-semiconductor (CMOS) microelectromechanical system (MEMS) technique to develop a low-power sensing system for VEGF detection. The monolithic interdigitated electrodes were used as the transducer; post-process etching and Au plating were also applied to the surfaces of the electrodes. Experimental results showed that the biosensor achieved a capacitive resolution of 28.3 fF and a sensing range of VEGF concentration from 1 to 1000 pg/mL [18]. While both studies [17,18] achieved highly sensitive detection of VEGF, complex fabrication procedures as well as an expensive setup were required to attain this level of sensitivity, and this may limit practical applications of such techniques. Hence, there is a need to produce a more cost-effective interdigitated sensor that is capable of detecting VEGF under less ideal operating conditions.

In this paper, we propose a very simple aptamer-based nanolayer grafting for VEGF detection on commercially available interdigitated sensors. In this work, the interdigitated fingers (90 μm wide) in our proposed device are spaced at 60 μm apart, and such distances can be easily produced under rural settings in terms of manufacturing and for upscaling purposes. DNA aptamers were used as a targeting moiety instead of conventional antibody; this is due to the fact that aptamers are more robust and resilient under a wider range of physiological conditions, and because these also have very high binding affinity to their targets [19]. In our very simple setup, we achieved detectable VEGF limits of 200 pg/mL (5 pM), while it has been suggested that VEGF in cancer patients can reach up to 310 pg/mL. Although the detection level does not reach the level of the commercial ELISA kit (such as 1 pg/mL of MBS355343, MyBioSource Inc., San Diego, CA, USA) [20], we found that our inexpensive setup was within the clinically relevant range [21] and that there was plenty of room for future optimization. Additionally, the relative cost-effectiveness makes the proposed approach a promising method for VEGF detection in practical point-of-care applications, especially in rural settings.

## 2. Methods and Materials

**Figure 1:**
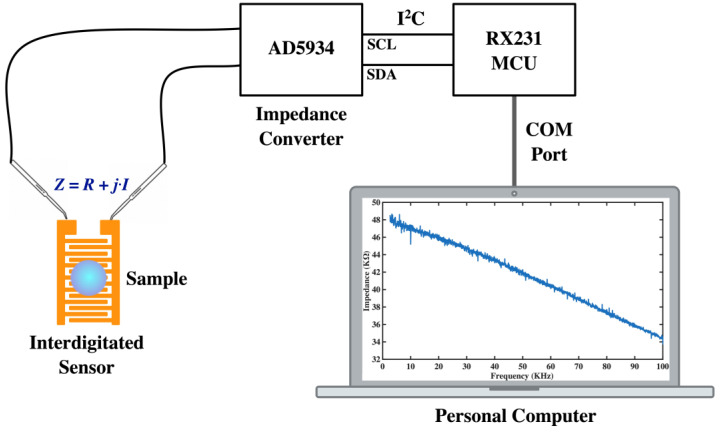
Configuration of the impedance measurement system used in this research.

In this study, we used the impedance analyzer AD5934 (Analog Devices Inc., Wilmington, MA, USA) [22] to measure the impedance. This analyzer is a high-precision impedance converter system that combines an on-board frequency generator with a 12-bit, 250 k sample per second (SPS) analog-to-digital converter (ADC). The frequency generator applies a sinusoidal-excitation signal to the external complex impedance at the specified frequency, and the on-board ADC samples the response signal from the impedance, which is then analyzed by the on-board digital signal processing (DSP) engine. The discrete Fourier transform (DFT) algorithm returns both real (denoted as *R*) and imaginary (denoted as *I*) parts of the impedance at each specified frequency along each sweep (ranging from 2.5 kHz to 100 kHz, with a 50-Hz separation between sweep frequencies). We used the RX231 microcontroller unit (MCU) (Renesas Electronics Corp., Koto-ku, Tokyo, Japan) [23] to control the impedance scanning and the data access on AD5934 via the inter-integrated circuit (I^2^C) bus. The impedance data acquired by the MCU is transported to a personal computer via the communication (COM) port and can be examined using the graphical user interface (GUI) application program (App) that was written in the C# language. The impedance spectroscopy can be shown in the form of magnitude versus frequency, or imaginary part versus real part, using the GUI App. The configuration of impedance measurement is depicted in Figure 1. The magnitude of impedance is derived by $\sqrt{R^{2}+I^{2}}$, the real part (*R*), imaginary part (*I*), and the magnitude values can be saved in a comma-separated values (CSV) file format using the developed App. In the illustrations for this paper, impedance (KΩ) represents the magnitude of the impedance, which is the imaginary part (*y* axis) versus the corresponding real part (*x* axis) in the Nyquist plot. The phase of impedance can be derived using $\tan^{-1}\left(I/R\right)$. As the magnitude or Nyquist plot is adopted in most articles, and the phase information lacks a scientific link to target concentration, the phase spectrum is omitted in this research.

**Figure 2:**
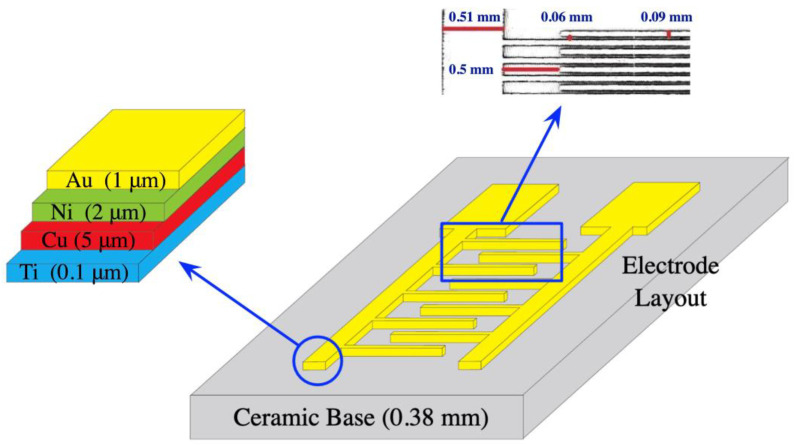
The structure of the interdigitated sensor.

The manufacturing procedure for interdigitated sensors typically involves the following steps [24]: First, the substrate (such as ceramic, silicon, glass, quartz, or crystalline polyethylene terephthalate) is cleaned and prepared to ensure a clean and uniform surface. Then, layers of metals (such as gold, platinum, nickel, copper, and titanium) are deposited on the substrate by sputtering, evaporation, or chemical vapor deposition to form the conductive surface of the interdigitated sensors. Next, photolithography is applied to the metal layers using a patterned mask and exposing them to ultra-violet (UV) light. This process chemically modifies the areas that are not covered by the mask, allowing the unexposed areas to be selectively removed in a subsequent etching step. The specific details of the manufacturing process may vary depending on the application and desired characteristics of the interdigitated sensor. However, these basic steps provide a general overview of the process, enabling mass production of interdigitated sensors. In this research, interdigitated sensors numbered 10856975, manufactured by Guangzhou MecartSensor Tech. Inc. (Guangzhou, Guangdong, China. Website: http://cn.global-trade-center.com/HotOffers/10856975.html, accessed on 19 May 2023) were utilized for all experiments. These sensors are cost-effective, with a price of less than 0.15 US dollars per piece under bulk orders. Figure 2 illustrates the structure of the interdigitated sensor used, where the conductive layers composed of titanium, copper, nickel, and gold are deposited on a ceramic substrate from bottom to top. This figure also demonstrates the thickness of each conductive layer and the geometric layout, including the gap between fingers, width of arms and fingers, and the separations between arms and fingers.

**Figure 3:**
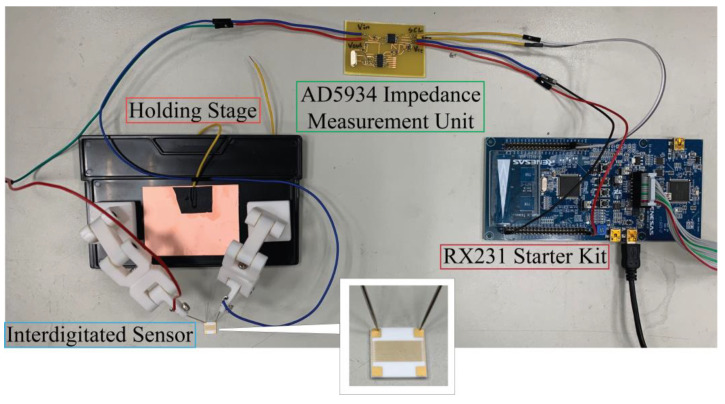
The customized setup for VEGF detection on our interdigitated sensors. A small aliquot of the protein was directly added to the surface in order to monitor the changes of impedance instantly.

Briefly, in our setup, the modified interdigitated sensor was mounted to a simple customized setup as a proof-of-concept as shown in Figure 3. The impedance measurement platform has been calibrated and tested using discrete components (510-KΩ resistor and 0.1-μF capacitor separately) according to the procedure suggested in the data sheet of AD5934 [22]. The discrete component tests were conducted before each experiment to assure the correctness of the followed impedance measurement. A single drop of protein solution was subsequently aliquoted directly onto the surface of the sensor carefully; the changes in impedance were immediately monitored using the RX231 starter kit which was subsequently connected to a computer workstation. All measurements were performed under normal ambient conditions to best mimic the environment in which handheld point-to-care devices are used. This is to demonstrate the ease of preparation of our simple setup that holds potential for future device system setups.

**Figure 4:**
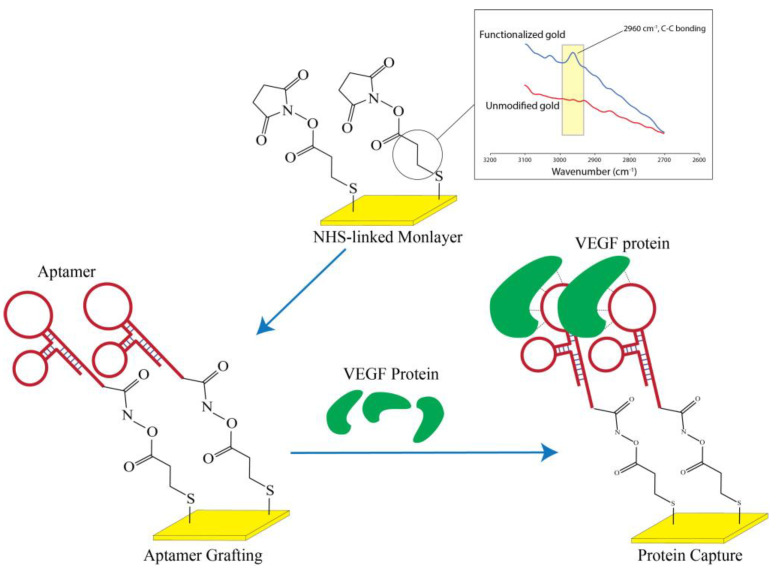
Depiction of the nanolayer aptamer grafting and protein capture for VEGF detection. The ATR-FTIR was also performed on the gold surface to validate the presence of the NHS grafting through the thiol group to the surface.

The surface modification and preparation of the nanolayer aptamer graft on the surface closely follows the previous work published from our group [13]; however, in the present case, the initial grafting relied on thiol-based linkages to the surface (Figure 4). In brief, 20 mM of 3-Mercaptopropanyl-N-hydroxysuccinimide ester (Sigma-Aldrich, Burlington, Massachusetts, USA) was prepared in a 50% EtOH solution and was directly added to the interdigital surface for two hours at room temperature under constant slow shaking. The thiol moiety would subsequently self-assemble onto the gold surface to present a distal NHS group. After the reaction, the surface was washed with copious amounts of methanol, ethanol, chloroform, and deionized water in sequential order and dried under a stream of nitrogen.

For protein biosensing intentions, 50 nM of DNA aptamer for VEGF 165 (Sequence: 5′ATACCAGTCTATTCAATTGGGCCCGTCCGTATGGTGGGTGTGCTGGCCAGATAGTATGTGCAATCA 3′) [25] was added to the NHS functionalized surface for a duration of 24 h at a pH of 8. After the incubation of the surfaces with the DNA, the interdigital sensors were washed with copious amounts of deionized water and dried under a stream of dry nitrogen and stored prior to use. Pertaining to the protein capture studies, both VGEF and PDGF were acquired from Novubios and were diluted to the apparent concentrations (5 and 50 pM) using PBS prior to dispensing protein drops directly onto the interdigitated surfaces for biosensing purposes using the setup as shown in Figure 3.

To examine the chemical profiling on the surface immediately after monolayer grafting, Attenuated Total Reflectance (ATR) was performed using JASCO FT/IR-4700 spectrometer with a Mercury Cadmium Telluride (MCT) detector that was cooled using liquid nitrogen during the analysis. Chemical signals from the gold surfaces were detected with a diamond prism at an angle of 45° by carefully aligning the gold region to the center of the detection window. Due to the implications from various NH species after aptamer grafting, we restricted our analysis solely on the gold surface passivated with NHS monolayer and examined the range from 2600 to 3500 cm^−1^ in order to elucidate the presence of aliphatic carbon (Figure 4).

## 3. Results

The objective of this study was to develop a cost-effective method for detecting the VEGF protein using readily available interdigitated sensors that are modified using surface chemistry and grafted with aptamers. The aptamer-VEGF complex has a negative charge, which alters the dielectric characteristics between the interdigitated electrodes. This alteration leads to a decrease in impedance when VEGF is captured by the sensor. Since the impedance consists of a capacitance component and the resulting imaginary part of the impedance varies with frequency, impedance measurements were taken from 2.5 kHz to 100 kHz to evaluate the concentration of VEGF. To ensure consistency in measurements, the ambient temperature is controlled at around 25 °C in all experiments.

**Figure 5:**
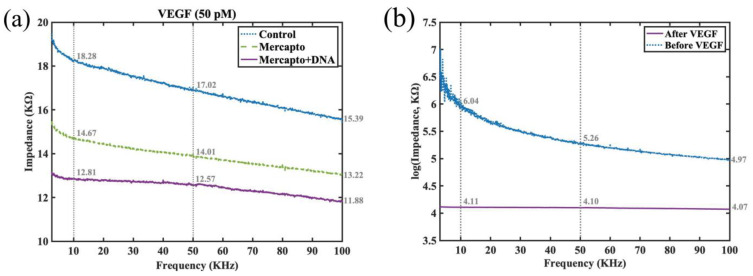
(**a**) Impedance-spectra testing for VEGF detection at 50 pM concentration under three different conditions: control (by the sensor without surface chemistry modification), mercapto (by the sensor with surface chemistry modification but no grafting of DNA aptamer), and mercapto plus DNA (using the sensor with surface chemistry modification and DNA aptamer grafting). (**b**) The impedance spectra before and after the VEGF protein samples were dropped on the sensor.

To examine the efficacy of surface-chemistry modifications and of DNA aptamer grafting for VEGF protein detection and selectivity, impedance-spectra testing was conducted for VEGF at a concentration of 50 pM under three distinct conditions using simple drop aliquots to the surface of the interdigital electrode. The examined conditions were as follows: control (using the sensor without surface chemistry modification), mercapto functionalized monolayer, and mercapto-plus-DNA (utilizing the sensor with surface chemistry modification and DNA aptamer grafting). It was expected that the mercapto-plus- DNA scenario would exhibit the lowest impedance in the spectra due to the capture of the negatively charged VEGF protein by the functionalized sensor. The experimental results shown in Figure 5a confirm that the functionalized sensor indeed exhibited the lowest impedance, while the unfunctionalized sensors without surface-chemistry modification presented the highest impedance for VEGF detection among the three scenarios. This experiment demonstrated the effectiveness of the DNA aptamer grafted interdigitated sensor for rapid VEGF protein detection. From the impedance values marked at 10, 50, and 100 KHz, it can be observed that there exists at least a 2.17 KΩ difference (the smallest is from 15.39 KΩ down to 13.22 KΩ at 100 KHz) in the impedance magnitude between the sensors with and without surface chemistry modification for 50-pM VEGF detection. In addition, there exists at least a 1.34 KΩ impedance decrease (with the impedance down form 13.22 KΩ to 11.88 KΩ at 100 KHz) after DNA aptamer grafting. Figure 5b shows the impedance change before and after the VEGF sample dropped on the interdigitated sensor. Please note, due to the huge difference in the impedance scale before and after the VEGF protein detection, the *y* axis has been converted to a logarithmic scale (base 10). From the marked values at the specific frequencies (10, 50, and 100 KHz), there exists an impedance difference of order 1 (from 4.97 to 4.07 at 100 KHz, which corresponds to a reduction of 78.87 KΩ of impedance magnitude) to order 2 (from 6.04 down to 4.11 at 10 KHz, which is a reduction of 108.36 KΩ of impedance magnitude).

**Figure 6:**
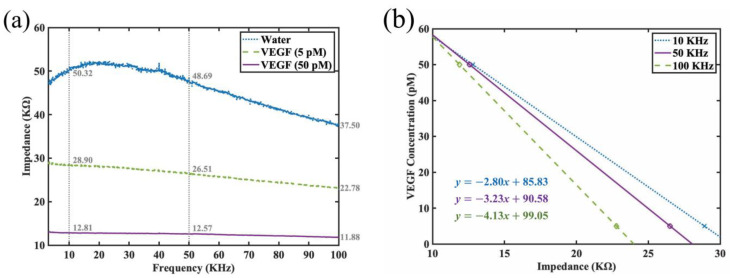
(**a**) Impedance spectra of the interdigitated sensor with surface chemistry modification and DNA aptamer grafting for three different conditions: water (buffered), VEGF detection at 5 pM concentration, and VEGF detection at 50 pM. (**b**) The regression lines for 10, 50, and 100 KHz that relate the impedance values (in KΩ) to VEGF concentrations.

To further evaluate the effectiveness of the functionalized sensor in VEGF detection, we also conducted further investigations into the impedance spectra for the interdigitated sensor with mercapto functionalized monolayer and DNA aptamer grafting under three conditions. The examined conditions included water (adjusted to pH 7), VEGF at 5 pM (quantified to approximately 200 pg/mL), and VEGF at 50 pM. The detection using water serves as a control for the detection experiments and is hypothesized to exhibit the highest impedance among the three conditions, while higher concentration of VEGF protein would result in lower impedance. The experimental results were as summarized in Figure 6a,b, with the impedance values being marked at 10, 50, and 100 KHz. In Figure 6a, it can be subsequently observed that as the functionalized sensor could bind to more VEGF proteins under higher concentrations, the negative charge in the protein would in turn modify the overall dielectric characteristics between the arms of the interdigitated sensor, thus leading to greater reduction in the impedance spectra. We noticed that this quenching of the impedance was even noticeable for 5 pM when compared to water as our control (from 50.32 KΩ down to 28.90 KΩ at 10 KHz), while the quenching of the impedance was most profound for 50 pM (from 28.90 KΩ down to 12.81 KΩ at 10 KHz). Hence, we did not find reason to increase the concentration further, and concentrations beyond 50 pM were not thought to be clinically significant. Similarly, our device was unable to monitor change in impedance in a stable fashion for concentration below 5 pM, and thus we did not attempt to reduce our concentration further. Figure 6b shows the linear regression lines that map the impedance values (in units of KΩ) to the estimated VEGF concentration (in units of pM), in which the dotted, solid, and dashed lines are the regression lines corresponding to the cases of 10, 50, and 100 KHz. From Figure 6a, it can be observed that the impedance values decrease gradually as the frequency increases for both 5-pM and 50-pM cases. Both impedance spectra can be considered to be approximately straight lines with minor negative but somewhat different slopes. The linear regression lines at other frequencies can be easily explored. To avoid visual confusion, only three regression lines are shown in Figure 6b.

**Figure 7:**
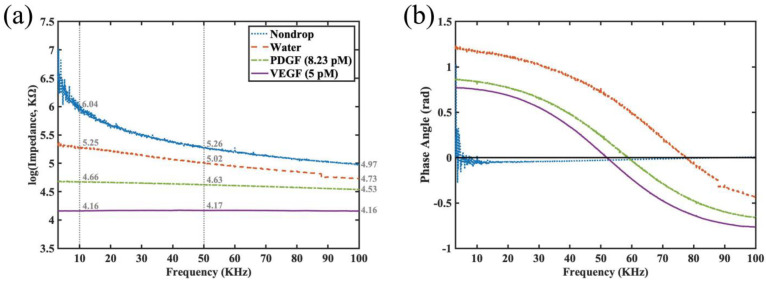
(**a**) Impedance spectra at logarithmic scale for the interdigitated sensor with surface chemistry modification and DNA aptamer grafting under four different conditions: nondrop (denotes the detection, as no sample is dropped on the sensor), water (buffered), PDGF detection at 8.23 pM concentration, and VEGF detection at 5 pM. (**b**) The corresponding phase spectra of (**a**).

Next, our group tested our sensor for detection of the presence of the non-specific second type of biomarker: platelet-derived growth factor (PDGF). It is important to note that both PDGF and VEGF are important signaling proteins involved in regulating cellular growth and angiogenesis and hence have significant clinical relevance. Both have distinct roles in physiological and pathological processes, and they target different receptors. PDGF is involved in cell growth and tissue repair, while VEGF is primarily responsible for promoting the growth of blood vessels [26]. Hence, it was the authors’ opinion that in the presence of PDGF, we will be able to evaluate whether non-specific interference could influence the impedance outcome of work. We also think this is an important consideration when performing clinical diagnosis using serum. The impedance spectra were measured under four different conditions: no sample, water, PDGF at 8.23 pM (approximately 200 pg/mL), and VEGF at 5 pM. Since the functionalized sensor is grafted with the DNA aptamer solely for the detection of VEGF protein, it is expected that the detected impedance for VEGF at 5 pM would be the lowest among the testing conditions. The experimental results are shown in Figure 7a, where the impedance values are presented in logarithmic scale due to the large dynamic range. In addition, the values at 10, 50, and 100 KHz are specifically marked to make it easier to comprehend the change on impedance in different conditions. The smallest difference between PDGF (8.23 pM) and VEGF (5 pM) is at 100 KHz. The practical impedance difference between 4.53 and 4.16 (in logarithmic scale of base 10) in fact reaches 19.43 KΩ. The impedance changes before and after PDGF protein exposure to the sensor were at least 59.44 KΩ (from 4.97 down to 4.53 at 100 KHz), whereas they were at least 78.87 KΩ (from 4.97 down to 4.16 at 100 KHz) for VEGF detection. From our data, we showed that even at our lowest limit of VEGF at 5 pM, our biosensor was able to discriminate among different biomarkers and was highly sensitive in terms of selectivity. The results were highly encouraging. Figure 7b demonstrates the corresponding phase spectra of Figure 7a, where the same line styles and colors are adopted for the same experiment condition in both subfigures. The phase spectra shown here are used to determine whether any differences exist for the cases with and without a sample dropped on the functionalized sensor. From Figure 7b, it can be observed that the phase spectra for the conditions with the dropped sample look very distinct from those without a dropped sample. It can also be appreciated that regarding the trend in the phase spectrum of water, PDGF and VEGF experiments were similar, i.e., the phase angle decreases gradually as the frequency increases. In addition, the phase angle is positive at low frequency and becomes negative at high frequency, which is similar to the results demonstrated in [27]. Because a scientific link between the phase angle and the sample concentration is lacking, specific marks like those appearing in Figure 7a are not shown in Figure 7b. Besides, the oscillation in the phase angle in the case of no sample dropped on the sensor at low frequency (below 15 KHz) may indicate more stable characteristics of the adopted interdigitated sensor when operating at frequencies higher than 15 KHz.

**Figure 8:**
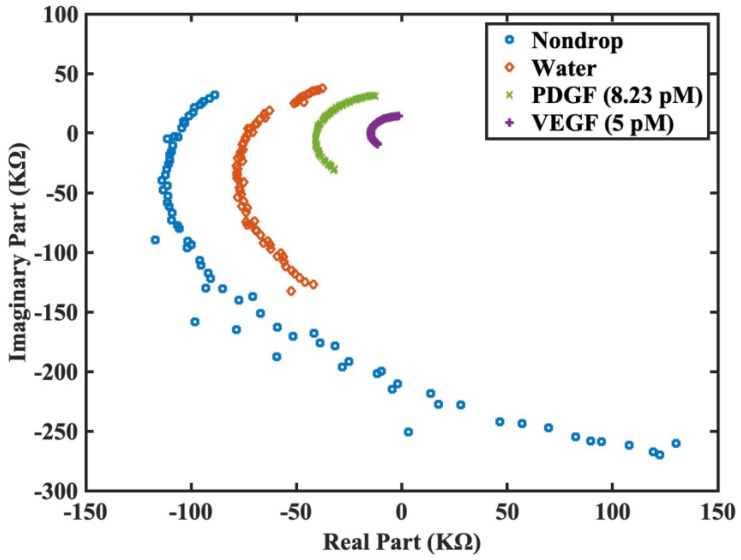
Impedance spectra presented in separate real and imaginary parts for the interdigitated sensor with surface chemistry modification and DNA aptamer grafting under four different conditions: nondrop (denotes the detection as no sample is dropped on the sensor), water (buffered), PDGF detection at 8.23 pM, and VEGF detection at 5 pM.

Thus, from Figure 5, Figure 6 and Figure 7, the proposed functionalized sensor was experimentally confirmed via impedance spectroscopy to be effective in its function, sensitivity, and specificity for VEGF detection. To further enhance the visualization of the derived impedance spectrum in an intuitive manner, we proposed the Nyquist plot presentation style in Figure 8. This style presents the real and imaginary parts of the spectrum on *x* and *y* axes separately for each frequency. Figure 8 utilizes the raw data from Figure 7 without the logarithmic scale. This illustration offers the advantages of intuitive comprehension and potential integration with machine learning techniques for intelligent VEGF detection, in which the real and imaginary impedance values of the Nyquist plot can be arranged to be the feature vector of the machine-learning classifier. Such a machine-learning-based approach was adopted in article [28] for the development of an impedimetric immunosensor on the detection of endometriosis.

It can be observed that most values of the real and imaginary parts of impedance are negative in the Nyquist plot (refer to Figure 8), which conflicts with the equivalent circuit models for interdigitated sensor summarized in [14] (especially the real parts, which should be positive). However, from the equivalent circuit model proposed in [29], the equivalent admittance for interdigitated sensor at angular frequency ω is as follows:

$$Y_{\mathrm{eq}}=\frac{1}{Z_{\mathrm{eq}}}=j\omega C_{\mathrm{sub}}+j\omega C_{p}+{\left(\frac{1}{j\omega C_{a}}+\frac{1}{G_{g}+j\omega C_{g}}\right)}^{-1}$$

where $Z_{\mathrm{eq}}$ is the equivalent impedance of the interdigitated sensor, $C_{p}$ and $C_{\mathrm{sub}}$ are the parasitic capacitances between interdigitated fingers and between substrates, $C_{a}$ represents the parasitic capacitance across the air, and $G_{g}$ and $C_{g}$ represent the conductance and capacitance via the sample, respectively. After mathematical manipulation on the above equation, the last term can be reformatted as follows.

$${\left(\frac{1}{j\omega C_{a}}+\frac{1}{G_{g}+j\omega C_{g}}\right)}^{-1}=\frac{\omega^{2}C_{a}C_{g}(C_{a}+C_{g}-G_{g})}{G_{g}^{2}+\omega^{2}{\left(C_{a}+C_{g}\right)}^{2}}+j\omega \frac{C_{a}C_{g}[G_{g}+\omega^{2}(C_{a}+C_{g})}{G_{g}^{2}+\omega^{2}{\left(C_{a}+C_{g}\right)}^{2}}$$

It can be observed that the real part of $Y_{\mathrm{eq}}$ contains the term $\left(C_{a}+C_{g}-G_{g}\right)$, which may lead to a negative value. The impedance $Z_{\mathrm{eq}}$ can be derived by taking the reciprocal of $Y_{\mathrm{eq}}$ and then multiplying the complex conjugate of $Y_{\mathrm{eq}}$ on the denominator, which implies that the real part of $Z_{\mathrm{eq}}$ is also the same sign as that of $Y_{\mathrm{eq}}$. Therefore, for the case of $Y_{\mathrm{eq}}$ with a negative real part, its corresponding impedance $Z_{\mathrm{eq}}$ also possesses a negative real part. From the experimental results shown in Figure 8, the equivalent circuit of the interdigitated sensor used in this research seems to be approximately the one shown in [29]. Please note that the equivalent circuit model for the interdigitated sensor is complicated, the parasitic values may deviate under different experimental conditions in different parts of the interdigitated sensor, and the model may also be distinct at a different frequency range [14]. Lastly, the data shown in the Nyquist plot (Figure 8) are all complex-valued and there is no impedance value that is purely resistive but negatively valued. The data points that are negative in the real part and are near the *x* axis still possess a relatively tiny imaginary part.

## 4. Discussion

We demonstrated a highly cost-effective approach for detecting VEGF using impedance spectroscopy with a surface monolayer functionalized interdigitated sensor. Commercially available interdigitated sensors were modified through simple but precise surface chemistry, and DNA aptamer was selected for VEGF protein detection. The platform for impedance spectroscopy, as shown in Figure 1 and Figure 2, was fabricated, and the processes of surface-chemistry modification and DNA aptamer grafting for VEGF detection were described using graphical schematics that are easy to follow (see Figure 4). Subsequent sensitivity experiments have been conducted, and the functionalized sensor was shown to possess the ability to detect VEGF at a concentration of 5 pM (Figure 5 and Figure 6). In order to assess the specificity of the functionalized sensor in VEGF detection, we compared the performance with the detection of non-specific PDGF. Finally, we also propose an alternative demonstration style for impedance spectra to enhance the intuitive visualization of the derived impedance spectrum, as depicted in Figure 8. Furthermore, this approach holds the potential to integrate the machine learning techniques for intelligent VEGF detection.

So far, as a proof-of-concept, we produced a rather simple and inexpensive interdigital sensor for the detection of clinically relevant biomarkers at sensitivities of up to 5 pM which could be quantified to approximately 200 pg/mL (molecular weight of VEGF 165: 40 kD). For a clinical setting, this concentration is technically at a mid-high range, for example, large scale meta-analysis had shown that the concentration of VEGF in blood serum would be in excess of 300 pg/mL for several major cancer types (breast, colon, and prostate) and even higher for others [30]. Hence, from an initial point of view, the lower limits of our device were within the threshold of clinical relevancy. In conjunction with this, we also decided to re-test a non-specific PDGF protein, and we were able to elucidate a stronger quenching of impedance on our surface modified sensors. This suggested that the nanolayer-surface modification was indeed sufficient and necessary to provide the intended sensitivity to the surface and the design of the biosensor was deemed successful in this context.

## 5. Conclusions

In this work, we presented a simple and inexpensive interdigitated biosensor incorporated with simple surface chemistry that can be quickly assembled as a viable biosensor in a nutshell. We showed that even without nanometer fabrication, we could achieve highly comparable detection of VEGF. We do acknowledge that the detection limit (LOD) for VEGF may be at the mid-upper range clinically (200 pg/mL), but it is important to note that we achieved this measurement on a micrometer-fabricated interdigitated device. Pertaining to the sensitivity concerns, we plan to address this accordingly in future efforts to optimize our device. In these efforts, we hope to further reduce the size of the fingers as well as the spacing distance and to observe how these changes will affect the overall sensitivity of the device. However, it is necessary to note that we plan to keep the size and spacing within the micrometer regime for the purpose of commercialization, especially for rural settings. Furthermore, we had already selected a very simple and mild chemical functionalization approach to graft aptamers onto the surfaces at the nanolevel, and this was highly appealing for upscaling purposes. It is in the authors’ opinion that instead of highly complicated devices, simple assembly based on sound chemistry and well-established protocols could effectively produce workable biosensing platforms that would suffice for most clinical applications. Our device easily fits this description.

## Data Availability

Not applicable.
